# Gastric Ulcer in Children

**Published:** 1913-02

**Authors:** 


Gastric Ulcer in Children.—Cackovic, *Archiv. fur klin. Chir.* reports 172 operative cases of gastric ulcer, 2.52% in children under 10, 7.55% in those under 15. All but two cases recovered.

